# PREDICTORS OF FINANCIAL BURDEN IN TWO SAMPLES OF ADULTS WITH ALZHEIMER’S DISEASE AND RELATED DEMENTIAS

**DOI:** 10.1093/geroni/igad104.3531

**Published:** 2023-12-21

**Authors:** Jordana Clayton, Eli Iacob, Nancy Aruscavage, Rebecca Utz, Katherine Supiano, Sara Bybee, Kara Dassel

**Affiliations:** University of Utah, Salt Lake City, Utah, United States; University of Utah, Salt Lake City, Utah, United States; University of Utah College of Nursing, Salt Lake City, Utah, United States; University of Utah, Salt Lake City, Utah, United States; University of Utah, Salt Lake City, Utah, United States; University of Utah, Salt Lake City, Utah, United States; University of Utah College of Nursing, Salt Lake City, Utah, United States

## Abstract

Compared to other older adults with chronic illness, individuals diagnosed with Alzheimer’s disease and related dementias (ADRD) incur much higher care costs at the end of life. The financial strain created by these high costs is experienced unevenly, as individuals who are low-income and minorities may have difficulty meeting out-of-pocket costs of care. Our objective was to examine the relationship between reported concern about being a financial burden to others and socioeconomic and demographic variables. To accomplish this, we conducted a mixed-methods study of two samples of individuals diagnosed with ADRD aged 31-89 (mean 63, n = 44) and 50-89 (mean 66, n=38) using two ordinal logistic regression models and qualitative analysis. The ordinal regression models indicate that the probability of reporting higher financial burden increases with lower age, higher education, and being widowed. One model shows income and employment as significant predictors of financial burden, particularly for the “forgotten middle” who have a high enough income not to qualify for social support programs but low enough income to experience strain from high out-of-pocket costs. In the qualitative analysis, we categorized 309 free responses from the second sample using the Financial Hardship Typology into concerns of material conditions / ability to pay (n=40), psychological distress about being a financial burden (n=28), and coping behaviors / limiting care to reduce costs (n=20). These results point to ways that social programs need to be improved to better support individuals with ADRD who are experiencing financial strain during the end-of-life.

